# Dihydroartemisinin-driven selective anti-lung cancer proliferation by binding to EGFR and inhibition of NRAS signaling pathway-induced DNA damage

**DOI:** 10.1038/s41598-024-62126-8

**Published:** 2024-05-22

**Authors:** Liu-Gen Li, Xing-Chun Peng, Zi-Yi Yang, Ning Han, Chang-Long Gou, Jun Shi, Li-Li Yu, Nan-Nan Chen, Ting-Ting Yu, Tong-Fei Li, Xian-Yu Li, Jun Hu

**Affiliations:** 1 Shiyan Key Laboratory of Natural Medicine Nanoformulation ResearchHubei Key Laboratory of Embryonic Stem Cell Research, School of Basic Medical Sciences, Hubei University of Medicine, Renmin road No. 30, Shiyan, 442000 Hubei People’s Republic of China; 2 Department of Pathology, Shenzhen Pingle Orthopedic Hospital (Shenzhen Pingshan Traditional Chinese Medicine Hospital), Shenzhen, 518118 Guangzhou Province People’s Republic of China; 3 Department of Pathology, Sinopharm DongFeng General Hospital, Hubei University of Medicine, Renmin road No. 30, Shiyan, 442000 Hubei People’s Republic of China; 4 Department of Ultrasound Medicine, Taihe Hospital of Shiyan, Hubei University of Medicine, Shiyan, 442000, Hubei People’s Republic of China; 5 Shenzhen Stomatology Hospital (Pingshan) of Southern Medical University, Shenzhen, 518000 People’s Republic of China; 6 Traditional Chinese Medicine Hospital, Dianjiang, Chongqing, 408300 People’s Republic of China

**Keywords:** Dihydroartemisinin (DHA), Lung cancer, EGFR, NRAS signaling pathway, DNA damage, Phytomedicine, Cancer, Biomarkers, Molecular medicine

## Abstract

Chemotherapeutic agents can inhibit the proliferation of malignant cells due to their cytotoxicity, which is limited by collateral damage. Dihydroartemisinin (DHA), has a selective anti-cancer effect, whose target and mechanism remain uncovered. The present work aims to examine the selective inhibitory effect of DHA as well as the mechanisms involved. The findings revealed that the Lewis cell line (LLC) and A549 cell line (A549) had an extremely rapid proliferation rate compared with the 16HBE cell line (16HBE). LLC and A549 showed an increased expression of NRAS compared with 16HBE. Interestingly, DHA was found to inhibit the proliferation and facilitate the apoptosis of LLC and A549 with significant anti-cancer efficacy and down-regulation of NRAS. Results from molecular docking and cellular thermal shift assay revealed that DHA could bind to epidermal growth factor receptor (EGFR) molecules, attenuating the EGF binding and thus driving the suppressive effect. LLC and A549 also exhibited obvious DNA damage in response to DHA. Further results demonstrated that over-expression of NRAS abated DHA-induced blockage of NRAS. Moreover, not only the DNA damage was impaired, but the proliferation of lung cancer cells was also revitalized while NRAS was over-expression. Taken together, DHA could induce selective anti-lung cancer efficacy through binding to EGFR and thereby abolishing the NRAS signaling pathway, thus leading to DNA damage, which provides a novel theoretical basis for phytomedicine molecular therapy of malignant tumors.

## Introduction

Chemotherapy is an essential strategy for lung cancer treatment^1–3^. Conventional chemotherapeutic agents such as cisplatin, paclitaxel, and methotrexate are cytotoxic and can directly destroy malignant cells by damaging DNA^4,5^, blocking intracellular protein synthesis^6,7^, and affecting nucleic acid metabolism thereof^8–10^, which are limited by the collateral damage to normal cells. Mutations in various pro-oncogenes (such as RAS, MYC, and SIS family genes) in lung cancer cells are among the important endogenous factors leading to its development^11–13^. Therefore, the exploration of novel anti-cancer agents with molecular targeting functions, which can also selectively inhibit lung cancer cells (low toxicity and high efficiency), is a significant entry point for the treatment of lung cancer at present. As reported, the expression of the NRAS was lower in normal lung cells, but significantly higher in lung cancer cells. NRAS is a classical oncogene, whose mutation can activate the cell cycle through the downstream ERK/MAPK signaling pathway^14,15^, resulting in malignant proliferation^16^. It has also been recently shown that NRAS mutations not only directly promote cell proliferation, but also block intracellular DNA damage^17–19^. Accordingly, NRAS mutations can lead to resistance of tumor cells to a variety of chemotherapeutic agents that damage intracellular DNA^20,21^.

Traditional Chinese Medicine (TCM) has been harnessed to treat many kinds of diseases, including microbial, parasitic infections, and malignant tumors, with many of the medications having chemotherapeutic effects^22^. Several herbal formulations and their active ingredients have been utilized for the prevention of cancer development or as an adjunct to conventional radiotherapy and chemotherapy due to their lesser side effects and toxicity^23,24^. However, as the drug mechanisms of the complicated active ingredients in Chinese herbal medicines have not been fully revealed, there is still a long way to go for their research. *Artemisia annua L*. is a crucial medical plant according to the record of the Handbook of Prescriptions for Emergencies (by Ge Hong of the Eastern Jin dynasty). Recent studies have indicated that artemisinin, derived from the natural plant *Artemisia annua* *L.* and awarded the Nobel Prize due to the antiplasmodial effect, has a significant inhibitory effect on a wide range of solid tumors^25^. Dihydroartemisinin (DHA) is an essential derivative of artemisinin and the active component of artemisinin metabolized by the liver in the body. In our previous work, DHA has also been proven to be an effective promoter of apoptosis and suppressor of lung cancer cell viability at low concentrations. Noticeably, DHA inhibits tumors with minimal collateral effects on normal cells of the body^24,26,27^. Nevertheless, the selective anti-cancer mechanism of DHA is still uncovered.

We previously found that treatment of lung cancer cells with DHA led to DNA damage and subsequent response (DDR)^28,29^. As mentioned above, NRAS mutations can inhibit DNA damage in cells. DHA enhances DNA damage by inhibiting the NRAS signaling pathway in lung cancer cells is still enclosed. Hence, in the present work, the proliferation rate of lung cancer cells (A549 and LLC) and normal lung cells were compared first. Then the expression of NRAS and associated molecules were evaluated in these two kinds of cells. The following experiments revealed that the DHA’s effects on the NRAS signaling pathway, DNA damage, and proliferation ability of lung cancer cells. Molecular docking and CETSA were applied to analyze the target binding of DHA and epidermal growth factor receptor (EGFR). On the contrary, reverse NRAS over-expression was conducted to demonstrate the causal links among DHA-induced NRAS inhibition, DNA damage, and anti-proliferation. The findings were displayed in the present work. The innovation and significance of selective anti-cancer therapy were discussed.

## Material and methods

### *Cell cultures and DHA treatment *in vitro

16 HBE cell line (16 HBE), Lewis cell line (LLC), and A549 cell line (A549) were purchased from the Cell Bank of Shanghai Institutes for Biological Sciences (Shanghai, China). 16 HBE are known as a kind of bronchial epithelial cells, which were used as a normal lung cell model. LLC and A549 are mice and human lung cancer cells. As such, these were employed as models of lung cancer cells. Both of them were cultured in the DMEM medium (Sigma-Aldrich, St Louis, USA) containing 10% fetal bovine serum (QmSuero/Qingmu Biotechnology, Wuhan, China) in a hygroscopic environment (5% CO2/95% air, 37 degrees centigrade). In the present work, 10 or 60 μg/ml concentrations of DHA, which is a kind of chemical compound (purchased from MERYER, 77,939–50-9, MERYER, Shanghai, China, 98% purity) were used to incubate LLC or A549 for 16–24 h. The chemical structure of DHA was presented in Fig. 3I.

### Cell proliferation and apoptosis assay

LLC or A549 were labeled by CFSE probe respectively and seeded onto 24-well plates with 2 × 10^5^ cells per well. The cells were treated using DHA as described before and harvested into special tubes for flow cytometry assay (cytoflex, Beckman Coulter, USA, Hubei University of Medicine) and confocal microscopy (FV3000RS, Olympus, Japan, Hubei University of Medicine). The attenuated decay of CFSE fluorescence represented the inhibitory proliferation. Alternatively, the cells were seeded onto 6-well plates with a density of 8 × 10^5^. The proteins were extracted after DHA treatment. The proliferation-associated molecules (PCNA and Ki67) were analyzed by the Western blotting (WB) technique. At the same time, the cells in the well were photographed by microscopy. Alternatively, the Annexin-V/7-ADD-labeled cells seeded in the 24-well plates (2 × 10^5^) were collected for the apoptosis using the flow cytometry.

### Bioinformatic analysis

For the analysis of Cdc25a, NRAS, γ-H2A.X, and CDK4 expression in lung cancer, the transcriptome data of differential gene expression analysis in Tumor, Normal, and Metastatic tissues (TNMplot) website (https://tnmplot.com/analysis/) was harnessed^29^, wherein the normalized expression was utilized for the gene expression. After the data were visualized, Cdc25a, NRAS, γ-H2A.X, and CDK4 transcriptome expression data in lung cancer tissues and normal tissues were screened and plotted as statistical histograms.

### DNA damage measurement

For investigation of the DNA damage, the WB technique was used to detect relevant molecules (p53 and γ-H2A.X). On the other hand, comet assay was harnessed to analyze the DNA double-strand breakage, whose protocol was referenced by our previous study^30^. The DHA-treated cells were suspended in PBS and mixed with low melting point agarose (LMPA). The mixture was then dripped onto a glass slide pre-coated with normal agarose gel. The agarose gel was electrophoresed at 20 V, 300 mA for 20 min, then lysed in an alkaline lysis solution and neutralized using tris-Hcl. Finally, Hoechst was applied to stain the nuclei of cells. The cells were imaged using fluorescence microscopy.

### NRAS signaling pathway assay

For the detection of the NRAS signaling pathway, the proteins within LLC or A549 were extracted and carried out to perform western blots (WB). The expression of upstream molecules (NRAS, p-ERK1/2) was detected first. Alternatively, the associated proteins (Cdc25A, CDK4, and CyclinD1) that regulate the cell cycle were detected by the WB technique as well.

### Over-expression and knockdown of NRAS

The NRAS plasmid was purchased from GeneCreate Biological Engineering Co., Ltd, Wuhan, China. The NRAS siRNA was purchased from the Genomeditech Biological Co., Ltd. LLC or A549 seeded in 24-well or 6-well plates were incubated with NRAS plasmid (siRNA) and Lipofectamine8000 for about 8–10 h. The lipo6000 and NRAS plasmid (siRNA)-contained medium was replaced by fresh medium after NRAS transfection. The successful over-expression or knockdown of NRAS was characterized by the increased or decreased NRAS expression in cells. The transfected cells were then treated with DHA. The associated measurements (qRT-PCR and WB) were conducted.

### Parameter settings for Flow cytometry

After cells were treated and harvested as mentioned above, they were filtered into special tubes for flow cytometry analysis. Each channel was adjusted to the appropriate voltage before collecting cells. Each sample was collected with at least 1 × 10^4^ LLC or A549 cells. FITC-Annexin-V, CFSE, and FITC-NRAS plasmid fluorescence were collected in the FITC channel. PI fluorescence was obtained in the PE channel. For the FITC channel, the excitation and emission wavelengths were 488 and 525 nm, respectively, and for the PE channel, the excitation and emission wavelengths were 561 and 585 nm, respectively.

### WB and Co-immunoprecipitation analysis

Proteins obtained from LLC or A549 were washed three times and lysed in Ripa buffer with protease inhibitor. Quantification of cell lysates was carried out by centrifugation and analyzed using the BCA assay kit. Aliquots of proteins were fractionated by SDS-PAGE and transferred to PVDF membranes, which were blocked with 4% bovine serum albumin and incubated with antibodies EGFR (18,986-1-AP, Proteintech, Wuhan, China), PCNA (bs-2006R, Bioss, Beijing, China), Ki67 (bs-23103R, Bioss, Beijing, China), p53 (bs-2090R, Bioss, Beijing, China), γ-H2A.X (bs-3185R, Bioss, Beijing, China), NRAS (10,724-1-AP, Proteintech, Wuhan, China), p-ERK1/2 (bs-3016R, Bioss, Beijing, China), CDK4 (11,026-1-AP, Proteintech, Wuhan, China), CyclinD1 (26,939-1-AP, Proteintech, Wuhan, China), cdc25a (55,031-1-AP, Proteintech, Wuhan, China), GAPDH (PMK053C, BioPM, Wuhan, China), β-tubulin (PMF181F, BioPM, Wuhan, China) overnight at 4 °C. Next, the protein bands were incubated with secondary antibody. For protein exposure, the membranes were cut horizontally first. The membrane was washed after this and then incubated with the antibody for β-tubulin, which was developed the next day. The original blots could be found in Figs. S5, S6 and S7. Regarding the absence of adequate length original blots, we provided the following explanations: there are molecular weight differences between the proteins. Therefore, the membranes were cut prior to hybridisation with antibodies to ensure that each protein could be properly exposed. For the co-immunoprecipitation (CO-IP) detection, the proteins were pulled down using the agarose beads and then incubated by the antibody of EGFR. The washed EGFR protein was treated with sample preparation buffer. WB was utilized to detect the EGF expression which combined with EGFR.

### Quantitative RT-PCR (qRT-PCR) analysis

Extracted total mRNA from LLC or A549 was quantified to 1000 ng with a nanoliter and then reverse transcribed to cDNA using the transcriptor cDNA synthesis kit (PC5801, TRUEscript RT MasterMix, Beijing, Aidlab). The primers (5′ to 3′) of CDK4, NRAS, and GAPDH used in the present work were as follows:

Forward (ACCCAGAAGACTGTGGATGG) and reverse (TTCAGCTCAGGGATGACCTT) were applied for mouse GAPDH amplification.

Forward (CCTGCCGGTTGAGACCATTA) and reverse (AGGGCCATCTGGTAGCTGTA) were applied for mouse CDK4 amplification.

Forward (GGGAGATACGCCAGTACCGAATGAA) and reverse (AGGGCATCAGTGCAGCTTACTACAT) were applied for mouse NRAS amplification.

Quantitative PCR was conducted using a Bio-RAD CFX Connect Optics Module and data were analyzed using Bio-RAD CFXmanager. Amplification of cDNA was performed using a SYBRGreen real-time PCR Master Mix kit (PC3301, Beijing, Aidlab).

### In vivo* anti-cancer efficacy and IHC assay*

Female C57BL mice at 4 weeks of age were subjected to LLC (1 × 10^6^ cells/100 μL in PBS). Lewis cell-bearing mice were randomly divided into 2 groups, one of which was given DHA (i.p., 10 mg/kg b.w., once every other day, 4 times in total). After continuous drug administration, the mice were sacrificed and tumor grafts were harvested. Paraffin sections of neoplastic tissue were dewaxed, rehydrated, and analyzed with IHC. Briefly, the paraffin sections were subjected to incubation in 4% hydrogen peroxide at room temperature for 20 min. Incubating paraffin sections with 3% BSA for 1 h, followed by incubation with primary antibody at 4℃ overnight and staining with secondary antibody. The expression of molecules was observed last. Animal handling and experimental procedures were in line with protocols approved by the Animal Care Committee at the Hubei University of Medicine. All inoculations and treatments were carried out under Nembutal anesthesia. We confirmed that all methods were performed in accordance with the relevant guidelines and regulations of the Animal Care Committee at the Hubei University of Medicine (2024 No. 5).

### Network pharmacology analysis

Relevant targets for lung cancer were obtained from the databases CTD (https://ctdbase.org/), PharmGKB (https://pharmgkb.org/), and OMIM (https://omim.org/). Alternatively, related targets of DHA active ingredients were collected from PubChem (https://pubchem.ncbi.nlm.nih.gov/), TCMSP (https://tcmspw.com/tcmsp.php), and Swiss Target Prediction (http://www.swisstargetprediction.ch/). The Wayne diagrams are used to show their overlaps. For protein–protein interaction (PPI) network analysis, these common targets were imported into the STRING (https://string-db.org/) database to generate a PPI network as referenced by a previous study^31^, which was constructed for TCM-protein interactions. The co-shared genes were then visualized using the Cytoscape program.

### Molecular docking assay

EGFR complex (PDB code: 3IKA) crystal structure was determined in the RCSB PDB protein database (https://www.rcsb.org). Processing of the EGFR with PyMOL was followed by Autodock analysis of its energetic combination with DHA. The multiple binding sites of DHA with EGFR were analyzed. The most favorable free energy was chosen according to the principles described in auto-dock vina (http://vina.scripps.edu) and visualized with PyMOL.

### Cellular thermal shift assay (CETSA)

CETSA experiments were conducted to verify the binding ability of DHA to the EGFR. The CETSA protocol was carried out according to our previous study^32^. Briefly, Lewis cells were processed with DHA for 24 h, washed with PBS, extracted the proteins, and divided into 6 equal portions. Heating was carried out at 46, 50, 54, 58, 62, and 66 ℃ for 3 min, followed by cooling at 4 ℃ for 3 min, respectively. Finally, western blotting was utilized to measure the thermal stability of proteins. Based on the degradation of proteins, a heat melting curve was plotted.

### Statistical analysis

Statistical differences among groups were assayed using One-way analysis of variance (ANOVA). The data of statistics were displayed by the mean ± standard deviation (SD). *p* values < 0.05 were considered to be statistically significant.

### Ethics approval and consent to participate

Animal handling and experimental procedures conformed to the protocols approved by the Animal Care Committee at the Hubei University of Medicine (2024 No. 5).

### Statement

The authors confirmed that the present study was reported in accordance with ARRIVE guidelines (https://arriveguidelines.org). Furthermore, the plant collection and use was in accordance with all the relevant guidelines.

## Results

### Lung cancer cells possessed a more rapid proliferation compared with normal lung cells

**Figure 1:**
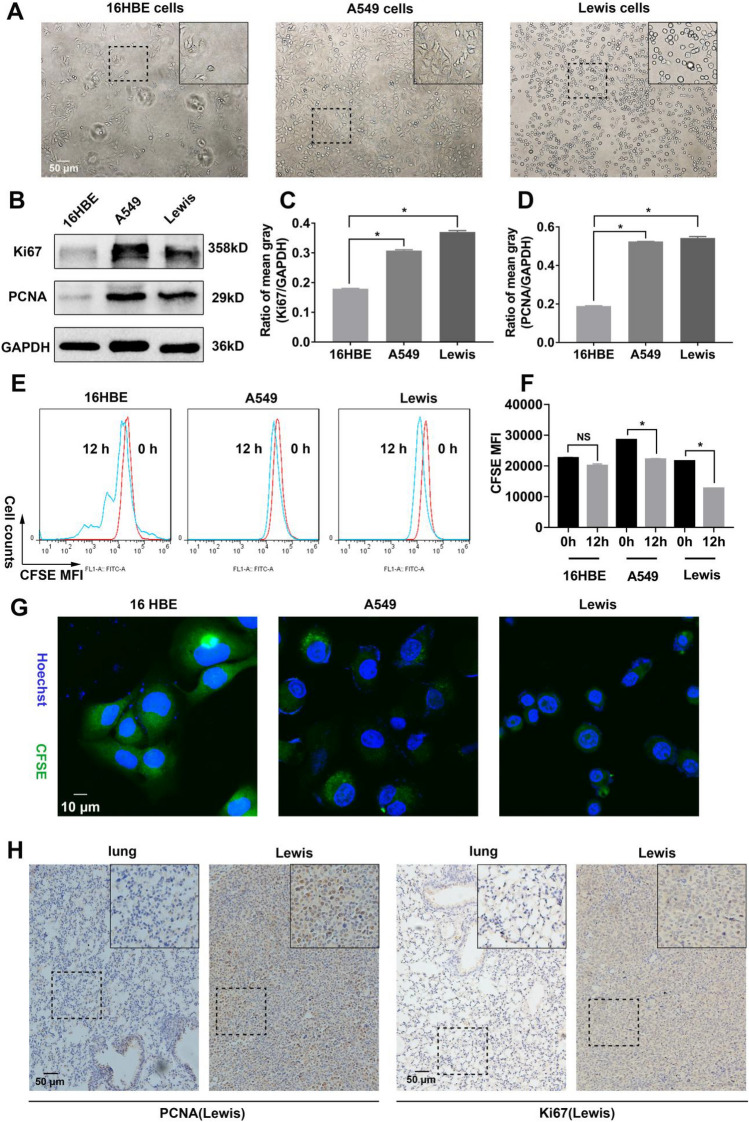
Malignant lung cancer cells presented rapid proliferation capability compared with normal lung cells. 16HBE, LLC and A549 were cultured for 24 h. (**A**) The cells were photographed by microscopy. 16 HBE had a slower proliferation rate than LLC and A549 thereof from the representative images. (**B–D**) The expression of Ki67 and PCNA, which are indicators of proliferation level, was assayed through WB. The mean gray of bands was quantitatively analyzed. (**E–F**) The cells were labeled with CFSE and assayed by flow cytometry. The decay of CFSE fluorescence suggested the proliferation of cells. (**G**) The cellular CFSE fluorescence was imaged by confocal microscopy. (**H**) The expression of PCNA and Ki67 in tumor grafts (Lewis cell-bearing mice) and lungs was assayed using immunohistochemistry (IHC). Values were means ± SD (n = 3, *p < 0.05).

First of all, the proliferation capability of malignant lung cancer cells and normal lung cells was investigated. These three kinds of cells were cultured for 24 h respectively. 16HBE, a kind of bronchial epithelial cells, had the slowest proliferation rate compared with LLC and A549 (mice and human lung cancer cells) as shown in Fig. 1A. Moreover, the expression of Ki67 and PCNA (proliferation-associated proteins) was lower than LLC and A549, suggesting that lung cancer cells proliferated rapidly (Fig. 1B–D). Additionally, the 16 HBE had the strongest CFSE fluorescence after 12 h in comparison with LLC and A549, indicating 16 HBE divided the slowest (Fig. 1E–G). On the other hand, the enhanced expression of PCNA and Ki67 was observed in cancer tissues compared with normal lungs in Lewis cell-bearing mice (Fig. 1H). Based on these data, we hypothesized that malignant lung cancer cells possessed the ability to proliferate and divide faster than normal lung epithelial cells.

### Lung cancer cells exhibited higher expression of NRAS

**Figure 2:**
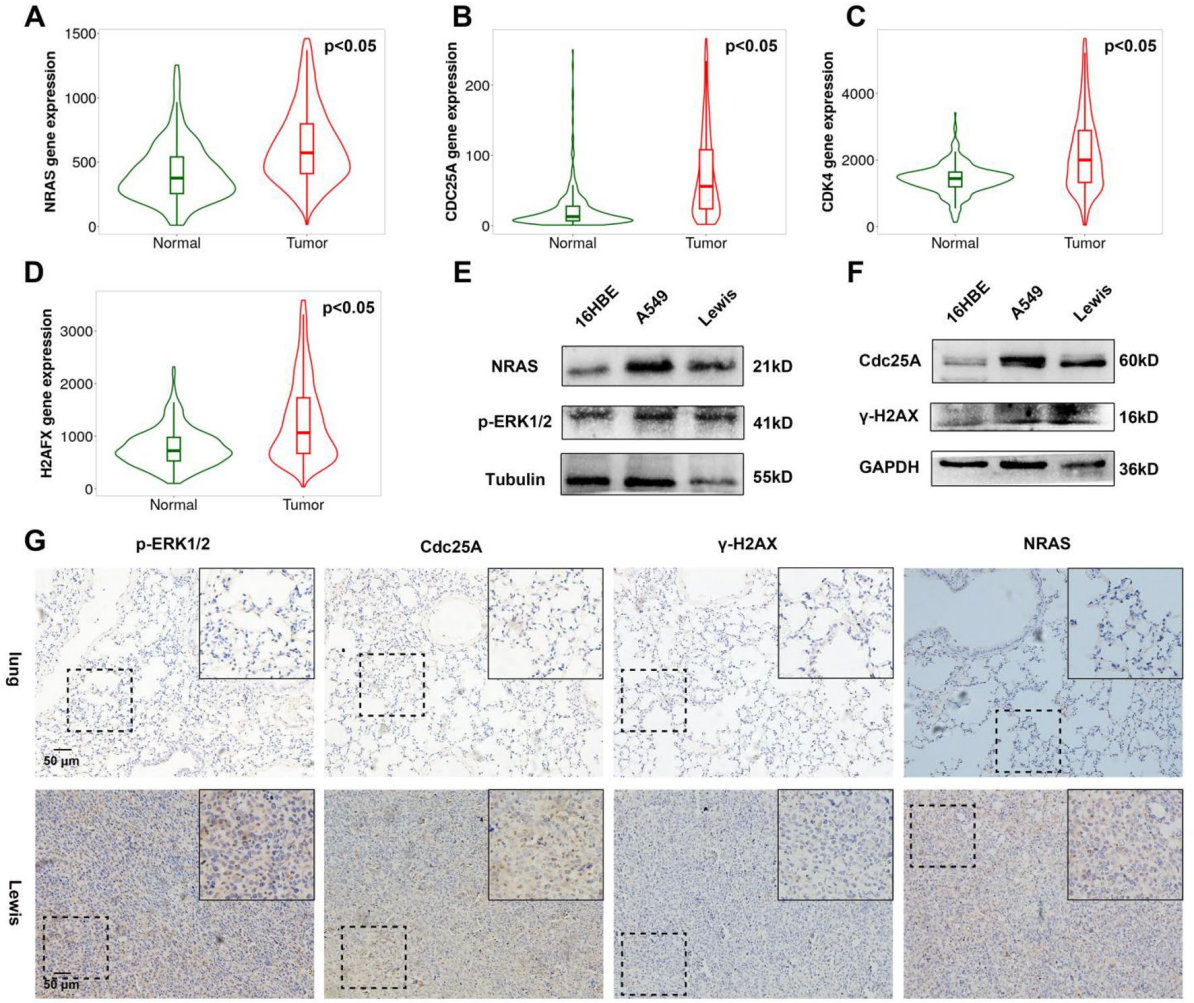
The expression of NRAS and p-ERK in Malignant lung cancer cells was higher than in normal lung cells. 16HBE, LLC and A549 were cultured for 24 h. (**A–D**) The normalized gene expression of NRAS, Cdc25a, CDK4 and γ-H2A.X in lung cancer tissues and normal tissues was analyzed and presented (*p < 0.05). The TNMplot database was utilized. (**E**) The expression of NRAS and p-ERK1/2 was analyzed through WB. (**F**) The DNA damage-associated molecules γ-H2A.X and proliferation-associated molecules Cdc25a were detected by WB. (**G**) The NRAS, p-ERK1/2, Cdc25A, and γ-H2A.X expression in tumor grafts and lung tissue was measured by IHC.

To clarify the underlying molecules and signaling pathways involved in the rapid proliferation of lung cancer cells, the associated oncogenes were explored thereupon. As mentioned before, NRAS is a classical oncogene, whose mutation can activate the cell cycle through the downstream ERK/MAPK signaling pathway, resulting in malignant proliferation. Transcriptome expression analysis from the GEPIA showed lung cancer tissues also exhibited higher NRAS, Cdc25a, CDK4, and γ-H2A.X expression (Fig. 2A–D). To analyze whether the downstream signaling pathway was activated, the expression p-ERK1/2 and Cdc25A, which were the critical molecules of NRAS^16^, was texted as well. As displayed in Fig. 2E–F, higher NRAS, p-ERK1/2, and Cdc25a expression could be observed in LLC and A549, confirming that the NRAS signaling pathway was activated in lung cancer cells. Interestingly but not surprisingly, the indicators of DNA damage (γ-H2A.X) varied little as presented in Fig. 2F. At the same time, the IHC assay showed that stronger expression of NRAS, p-ERK1/2, and Cdc25a was observed in lung cancer tissues compared with in normal lungs (Fig. 2G). Correspondingly, the indicators of DNA damage (γ-H2A.X) slightly intensified in tumor grafts (Fig. 2G). These findings indicated that NRAS signaling was a crucial carcinogenic factor, which probably worked by facilitating cell proliferation in lung cancer.

### DHA inhibited proliferation of lung cancer cells and exhibited anti-cancer efficacy

**Figure 3:**
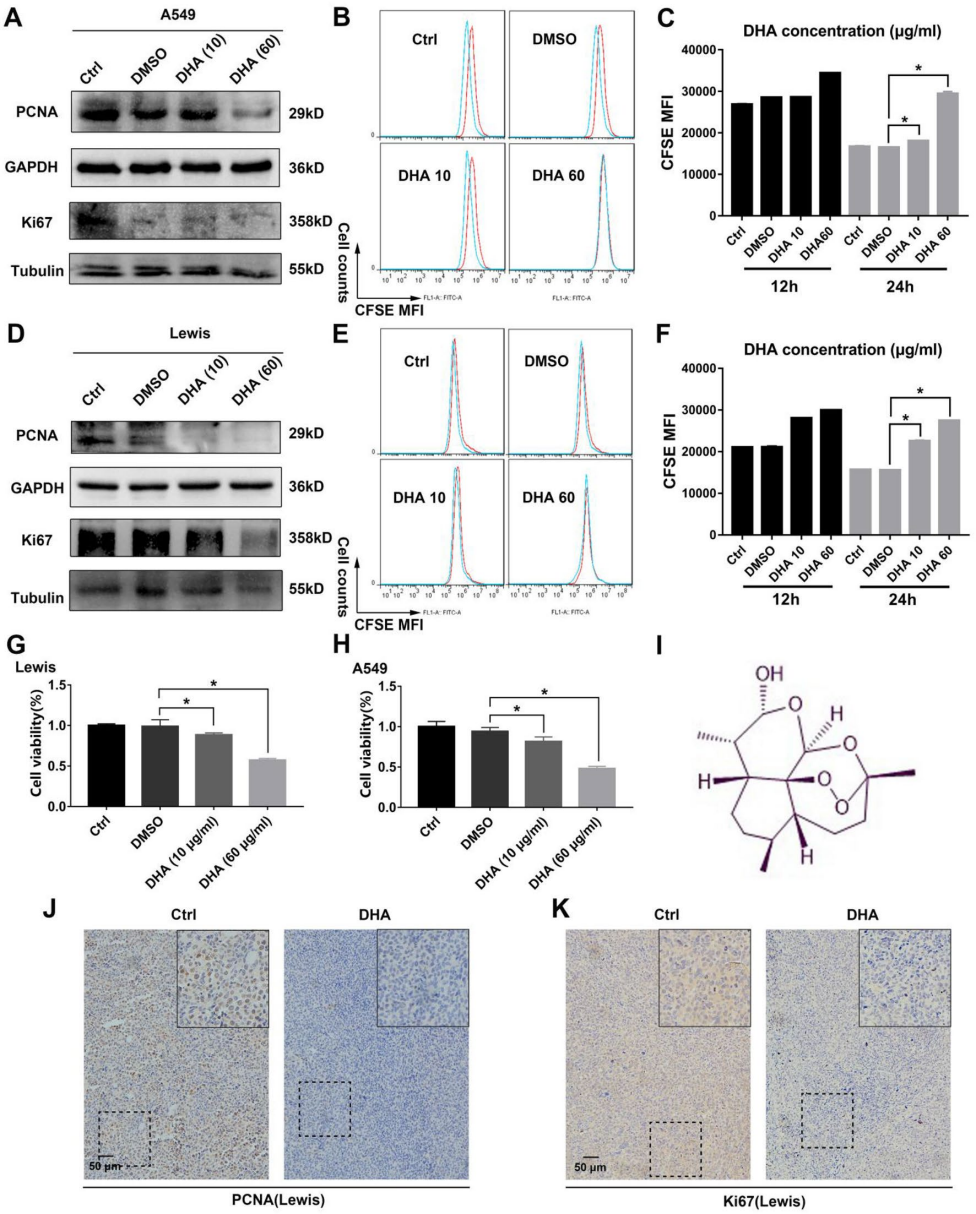
DHA significantly inhibited the proliferation of lung cancer cells. A549 and LLC were treated with DHA (10 or 60 μg/ml) for 24 h. DMSO treatment served as control. (**A**) The molecules (PCNA and Ki67) of proliferation were measured through WB in A549. (**B–C**) The A549 were labeled with CFSE, whose fluorescence decay (12 and 24 h) was detected by flow cytometry. **(D**) The molecules of proliferation were measured through WB in LLC. **(E–F**) The LLC were labeled with CFSE, whose fluorescence decay (12 and 24 h) was detected by flow cytometry. (**G**) The cell viability of LLC treated by DHA was assayed by CCK-8. (**H**) A549 cells’ viability was measured with CCK-8. (**I**) The chemical structure of DHA was presented. (**J–K**) The expression of PCNA and Ki67 in cancer grafts of DHA-treated tumor-bearing mice was detected using IHC. MFI: Mean fluorescence intensity. Values were means ± SD (n = 3, *p < 0.05).

**Figure 4:**
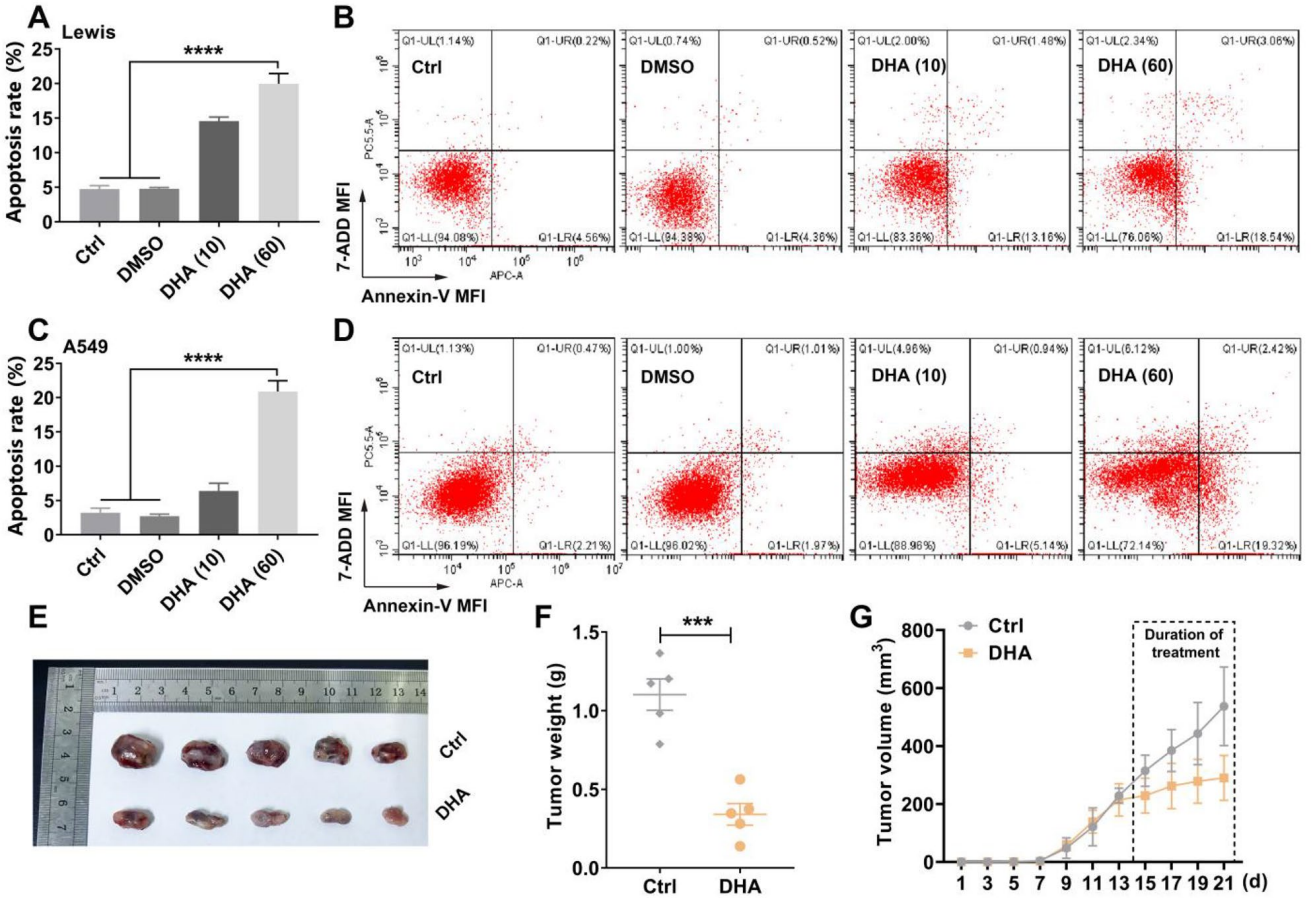
DHA promoted apoptosis and exhibited anti-cancer efficacy in tumor-bearing mice. (**A–D**) A549 and LLC were treated with DHA (10 or 60 μg/ml) for 24 h. DMSO treatment served as control. The cells were stained with Annexin-V and 7-ADD. The apoptosis rate was analyzed using flow cytometry. MFI: Mean fluorescence intensity. Values were means ± SD (n = 3, ****p < 0.0001). (**E–G**) The LLC-bearing mice were treated by DHA (i.p., 10 mg/kg, once every other day, 4 times in total). The size of tumor grafts was photographed at the end of the treatment (E). The tumor weight was recorded (F). The tumor volume was monitored during the treatment (G). Values were means ± SD (n = 5, ***p < 0.001).

Previously, we have demonstrated that DHA (Chemical structure displayed in Fig. 3I) had a selective anti-cancer function^29,33^, wherein the underlying mechanism had not been completely elucidated. We postulated whether its selective anti-lung cancer effect might be attributed to its action on NRAS signaling. To corroborate this hypothesis, the effect of DHA on the proliferation capacity of lung cancer cells was examined first. In A549, DHA treatment significantly suppressed the proliferation of cells, as evidenced by decreased expression of PCNA and Ki67 and inhibitory decay of CFSE fluorescence, which was particularly pronounced at 24 h (Fig. 3A–C). Consistently, in addition to A549 cells, the results in LLC also revealed proliferation was blocked in response to DHA treatment. (Fig. 3D–F). In addition, the cell viability of A549 and LLC was dampened in response to DHA treatment (Fig. 3G–H), further indicating the DHA’s proliferation inhibitory effect. The in-vivo relevance of the in-vitro findings was validated on LLC-bearing mice. DHA treatment significantly suppressed PDNA and Ki67 expression as presented in Fig. 3J–K. Moreover, we also explored the DHA’s anti-cancer efficacy in vitro and in vivo. As presented in Fig. 4A–D, The apoptosis rate of LLC and A549 increased dramatically in response to DHA. In consistency, the growth of tumor grafts was inhibited in the presence of DHA, as confirmed by the reduced tumor size, volume, and weight (Fig. 4E–G). Notably, the cell viability of 16HBE was slightly reduced with the treatment of DHA (Fig. S1), and the body weight of tumor-bearing mice treated with DHA varied little, indicating that DHA had low side effects on the normal tissues. (Fig. S2). Collectively, this proof suggested DHA could effectively inhibit the proliferation of lung cancer cells and bring about anti-cancer efficacy.

### DHA blocked NRAS signaling pathway and promoted DNA damage

**Figure 5:**
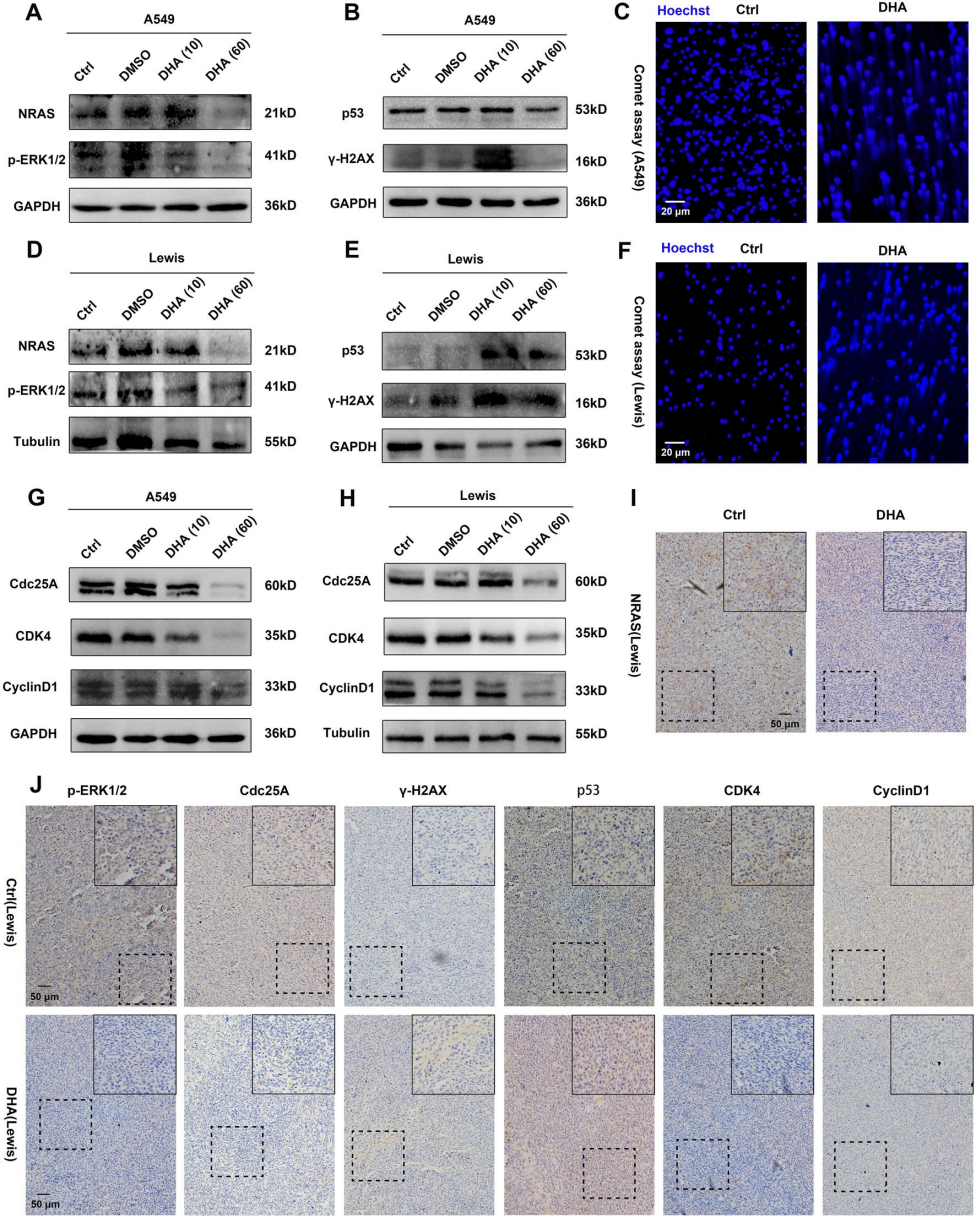
DHA blocked the NRAS signaling pathway and triggered DNA damage in lung cancer cells. A549 and LLC were treated with DHA (10 or 60 μg/ml) for 24 h. DMSO treatment served as control. (**A, D**) The expression of NRAS, p-ERK1/2 in A549 and LLC was detected by WB. (**B, E**) The indicators of DNA damage (p53 and γ-H2A.X) in A549 and LLC were assayed through WB. (**C, F**) Comet assay was applied to analyze the DNA double-strand breakage in A549 and LLC. (**G, H**) The biomarkers of the cell cycle (Cdc25A, CDK4, CyclinD1) in A549 and LLC were analyzed with WB. (**I–J**) The expression of NRAS, p-ERK1/2, Cdc25A, CDK4, CyclinD1, p53, and γ-H2A.X in tumor grafts was detected by IHC.

To further uncover whether DHA could affect the NRAS signaling pathway, we proceeded with the following experiments. As expected, DHA was found to down-regulate NRAS and p-ERK1/2 expression in A549 and LLC, suggesting NRAS signaling could be blocked by DHA (Figs. 5A, D, S3). As mentioned before, NRAS mutations can inhibit DNA damage in cells. We have demonstrated DHA could inhibit NRAS signaling. In that case, could DHA enhance DNA damage thereof? Thus, we next assessed the DNA damage in the lung cancer cells.

As shown in Fig. 5B, E, the biomarkers of DNA damage (p53 and γ-H2A.X) up-regulated in the presence of DHA in A549 and LLC. Interestingly, maybe when the DHA concentration reached 60 ug/mL, at this time, because the damage caused by the DHA to the cell was so great, the cells were completely dead, the γ-H2AX could no longer feedback expression in time, thereby showing down-regulation. Moreover, the results of the comet assay indicated DHA treatment promoted DNA double-strand breakage in lung cancer cells (Fig. 5C, E). Serious DNA damage usually leads to the arrest of the cell cycle so that the injured cells can be repaired timely^34^. To evaluate deeper the change of cell cycle in DHA-treated lung cancer cells, the molecules (Cdc25A, CDK4, and CyclinD1), which could regulate the process of the cell cycle, were assayed first. Treatment with DHA obviously decreased the expression of Cdc25A, CDK4, and CyclinD1, which mainly facilitate the cell cycle to the S phase from the G1 phase (Figs. 5G–H, S3). The arrest of the cell cycle might be the important contributor where DHA inhibited the proliferation of lung cancer cells. In agreement with the in vitro findings, the expression of NRAS, p-ERK1/2, CDK4, Cdc25A, and CydlinD1 was inhibited in tumor grafts with the treatment of DHA (Fig. 5I–J). However, the molecules of DNA damage (p53 and γ-H2A.X) up-regulated (Fig. 5J). Taken together, these findings were crucial evidence that DHA could impair the NRAS signaling pathway, thereby promoting DNA damage and arresting the cell cycle, which may be the mechanism by which DHA exhibited a selective anti-proliferation effect in lung cancer cells.

### DHA was the direct depressive inhibitor of EGFR

**Figure 6:**
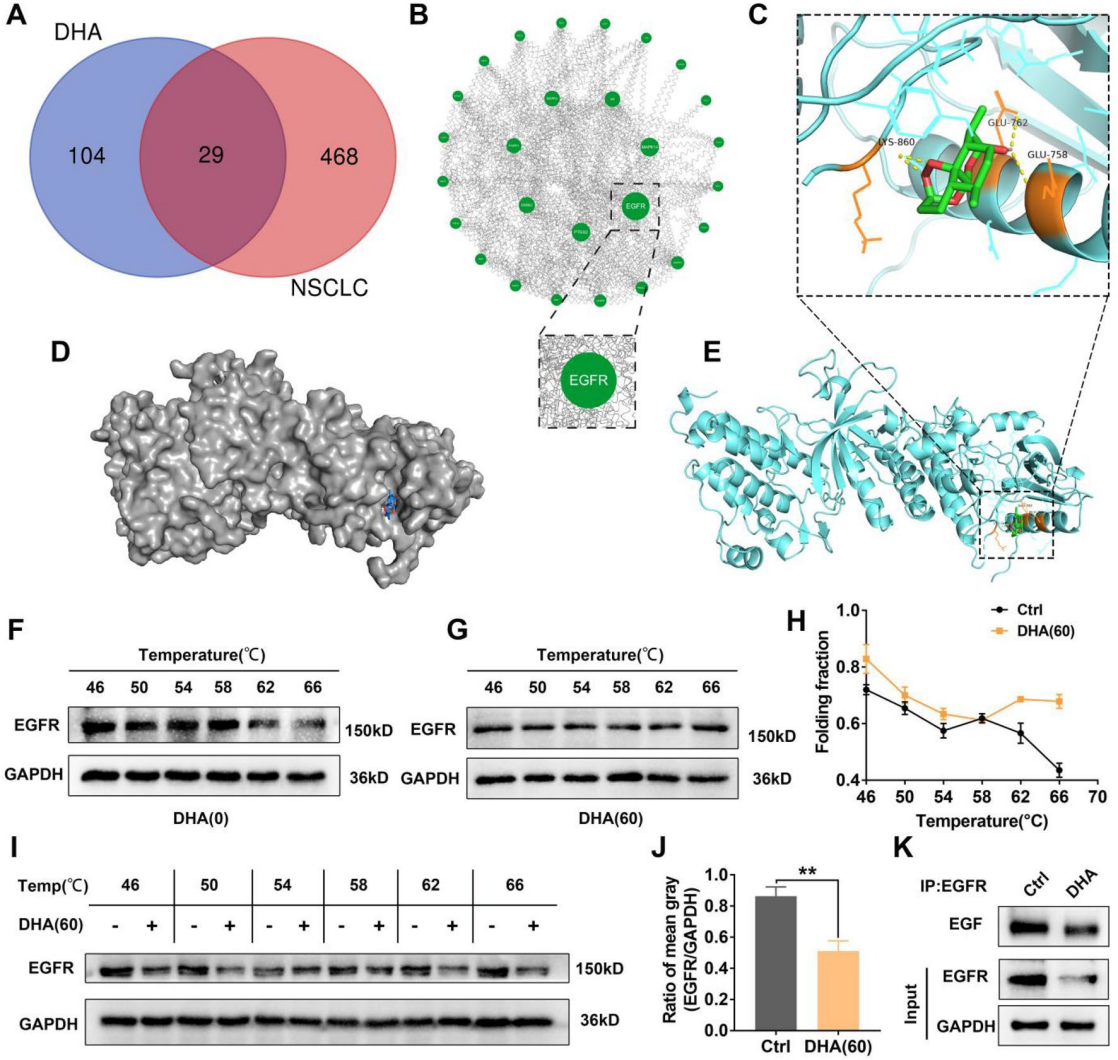
DHA could bind to EGFR and drive the suppressive function. (**A**) Venn diagram showing the overlap of DHA and Non-small cell lung cancer (NSCLC). (**B**) The protein interaction network diagram was constructed, wherein EGFR was the most significant interacting core target. (**C–E**) The crystal structure of DHA and EGFR and the molecular docking data were presented. The detailed information was presented in Table S1. (**F–H**) The targeting effect of DHA (60 μg/ml) on EGFR was analyzed by CETSA, wherein the expression of EGFR was detected by WB. The thermal solubility curve was displayed. (**I**) The expression of EGFR in DHA-treated lung cancer cells (60 μg/ml) was assayed by WB. (**J**) The mean gray of EGFR bands was analyzed and presented. Values were means ± SD (n = 5, **p < 0.01). (**K**) The EGF expression, which combined with EGFR, in the LLC was detected by the CO-IP.

To explore in depth why DHA inhibits NRAS signaling driving DNA damage, the network pharmacology analysis was carried out on the relationship between DHA and lung cancer. As a result, an interaction between DHA and lung cancer was found as presented by a Venn diagram (Fig. 6A), in which there were up to 29 interaction targets. Further protein–protein interaction (PPI) analysis showed that the core target of interaction was primarily centered on EGFR as displayed by a protein network diagram (Fig. 6B). For this reason, molecular docking was employed to analyze the interaction between DHA and EGFR. As presented, the crystal structure of EGFR was acquired from the protein database (PDB code:3IKA). The structure of DHA was proceeded using PyMOL. The detailed multiple binding sites of the molecular docking were listed in Table S1. The results indicated that there is a powerful H-bond interaction between DHA and the binding sites (GLU-762, GLU-758, LYS-860) of EGFR (Fig. 6C–E). Hence, the above data were strong indications towards a direct indication of DHA with EGFR.

Notably, the results based on molecular docking alone are not sufficient to account for the real binding of DHA to EGFR due to the limitation of the length of the EGFR crystal structure. Consequently, in a further step, proteins from DHA-treated lung cancer cells were subjected to Cellular Thermal Shift Assay (CETSA) to directly verify the binding ability of DHA to EGFR. EGFR in lung cancer cells became more difficult to degrade and its thermal solubility curve shifted significantly to the right After adding DHA (Fig. 6F–H). What’s more, EGFR expression was inhibited in lung cancer cells with the DHA treatment (Fig. 6I–J). The above outcomes suggested that DHA may be a direct inhibitor of EGFR. To validate the decrease in the function of EGFR after DHA binding, CO-IP was carried out to pull down EGFR, on which bound EGF was detected. As displayed in Fig. 6K, DHA treatment reduced the EGF binding on the EGFR. In a word, NRAS signaling blockage by DHA results from a discovery that has not been reported before regarding its ability to bind to EGFR proteins and drive the suppressive effect.

### Over-expression of NRAS abated DHA-induced DNA damage

**Figure 7:**
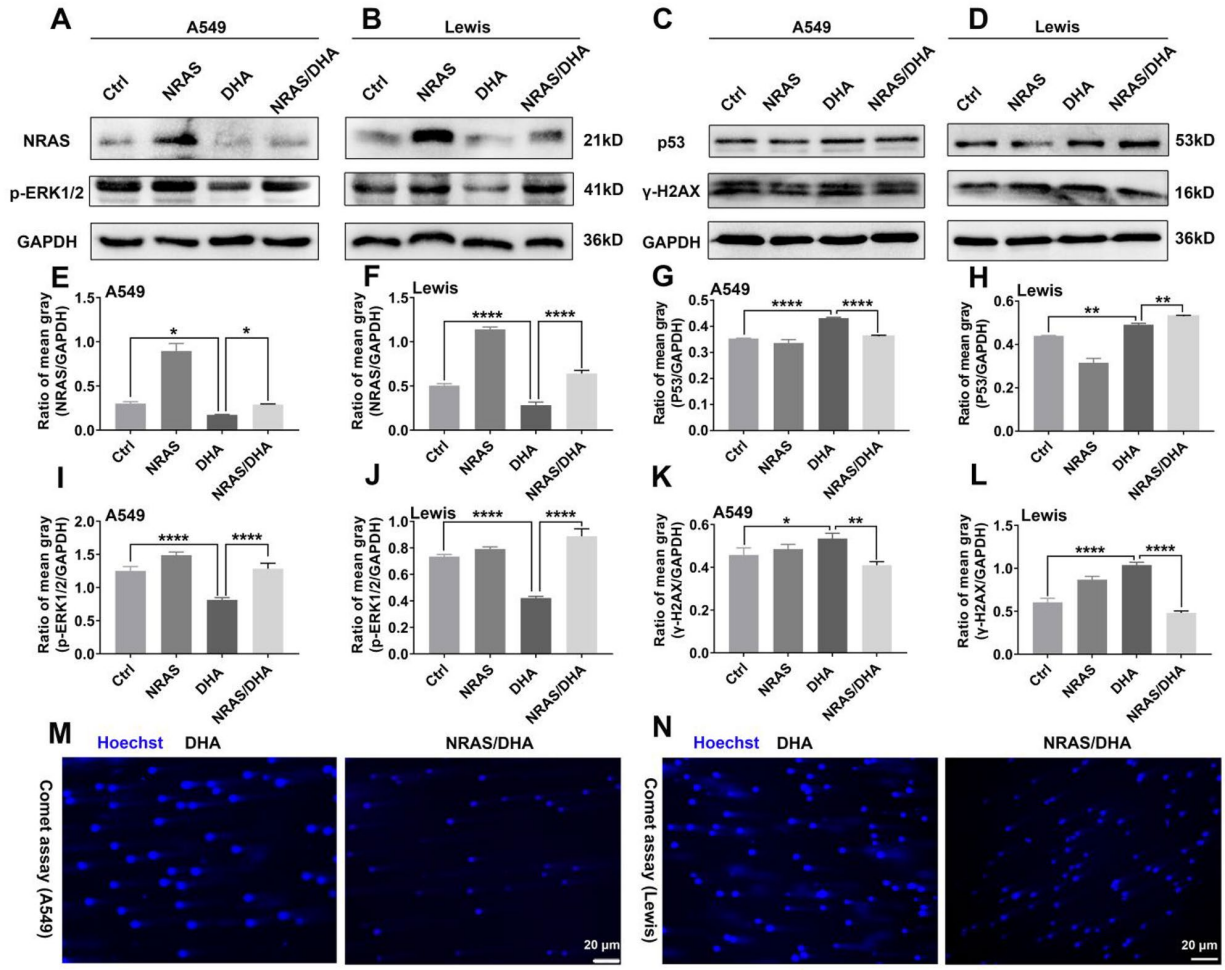
NRAS over-expression weakened DHA-introduced DNA damage of lung cancer cells. A549 and LLC were transfected by NRAS plasmid first, and then incubated with DHA (60 μg/ml). (**A–B**) The expression of NRAS and p-ERK1/2 in A549 and LLC was measured with WB. (**C–D**) WB revealed that over-expression with NRAS plasmid reduced DNA damage induced by DHA. (**E–L**) The mean gray of bands was quantitatively analyzed and presented. (**M–N**) Comet experiments suggested that NRAS plasmid recovery impaired DHA-triggered DNA double-strand breakage. Values were means ± SD (n = 3, *p < 0.05, **p < 0.01, ***p < 0.001, ****p < 0.0001).

For establishing the causal connection between DHA-mediated inhibition of the NRAS signaling pathway and the anti-proliferation effect from DHA, then NRAS over-expression was conducted in lung cancer cells. The results of NRAS and p-ERK1/2 expression in A549 and LLC demonstrated that the over-expression of NRAS was successful (Fig. 7A–B, E–F, I–J). Importantly, NRAS plasmid recovery abated DHA-induced DNA damage in lung cancer cells, as confirmed by decreased expression of p53 and γ-H2A.X (Fig. 7C–D, G–H, K–L). Another piece of evidence suggestive of reduced DNA damage with NRAS and DHA co-treatment was the weaker comet tail in A549 and LLC (Fi.g 7 M-N). Further findings of CDK4, and Cdc25A expression indicated NRAS over-expression re-activated process of the cell cycle of G1 phase to S phase (Fi.g 8B, C, F, G, J, K). These results gave us proof that DHA triggered DNA damage and arrested the cell cycle by blocking the NRAS signaling pathway in lung cancer cells.

### Over-expression of NRAS weakened DHA-mediated anti-lung cancer proliferation

**Figure 8:**
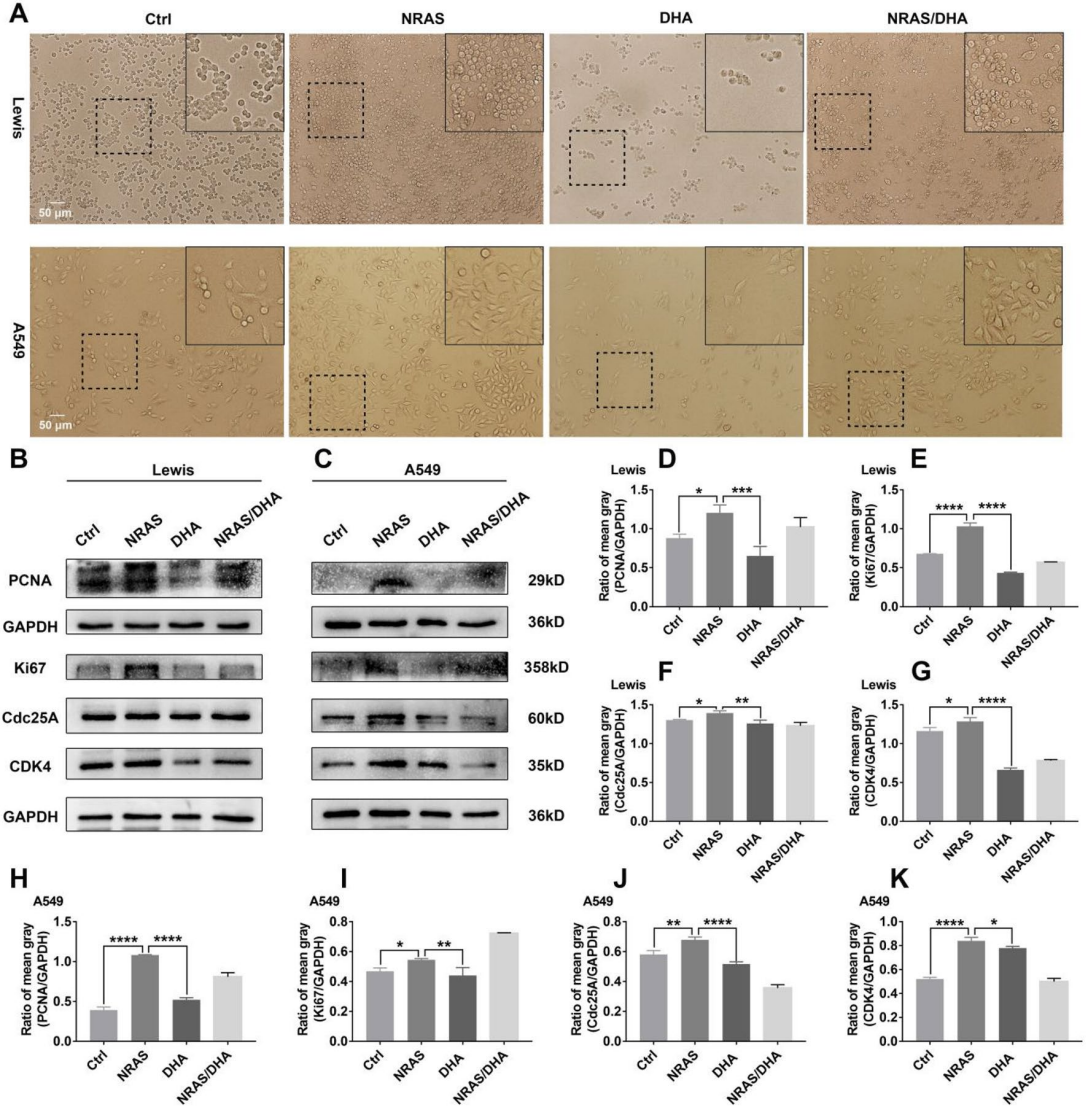
Over-expression of NRAS abated DHA-mediated suppression of proliferation in lung cancer cells. A549 and LLC were transfected by NRAS plasmid first, and then incubated with DHA (60 μg/ml). (**A**) The images of cell proliferation were captured using microscopy. (**B–C**) The expression of Ki67 and PCNA, which are indicators of proliferation, was enhanced in the presence of NRAS plasmid and DHA. The expression of CDK4, and cdc25a was up-regulated by when co-addition of NRAS plasmid and DHA. (**D–K**) The mean gray of bands was quantitatively analyzed and presented. Values were means ± SD (n = 3, *p < 0.05, **p < 0.01, ***p < 0.001, ****p < 0.0001).

Given that Over-expression of NRAS abated DHA-induced DNA damage and cell cycle arrest. Naturally, we concluded that this NRAS recovery effect, whereby the cell cycle arrest was diminished, would similarly render cell proliferation compromised. Hence, we proceeded to examine in depth the results of the over-expression of NRAS combined with DHA treatment on cell proliferation. As depicted in Fig. 8A, NRAS over-expression encouraged cell proliferation even if DHA treatment. Alternatively, the proliferation-associated proteins were also detected in NRAS plasmid and DHA-treated lung cancer cells (LLC and A549). The results showed expression of Ki67 and PCNA revitalized with the treatment of DHA and NRAS plasmid compared with DHA treatment alone (Fig. 8B, C, D, E, H, I), suggesting that recovery of NRAS molecule abolished DHA-driven anti-lung cancer proliferation. Additionally, DHA exhibited little effect on the downstream molecules of NRAS when the NRAS of the LLC was knocked down, as characterized by the slight variations of NRAS, PCNA, and CDK4 expression in the DHA-treated LLC with NRAS siRNA transfection (Fig. S4), which also indicated the critical role of NRAS. Consequently, all of these data recommended that DHA-mediated anti-lung cancer effect through blocking NRAS signaling and thereby inducing DNA damage.

## Discussion and conclusion

**Figure 9:**
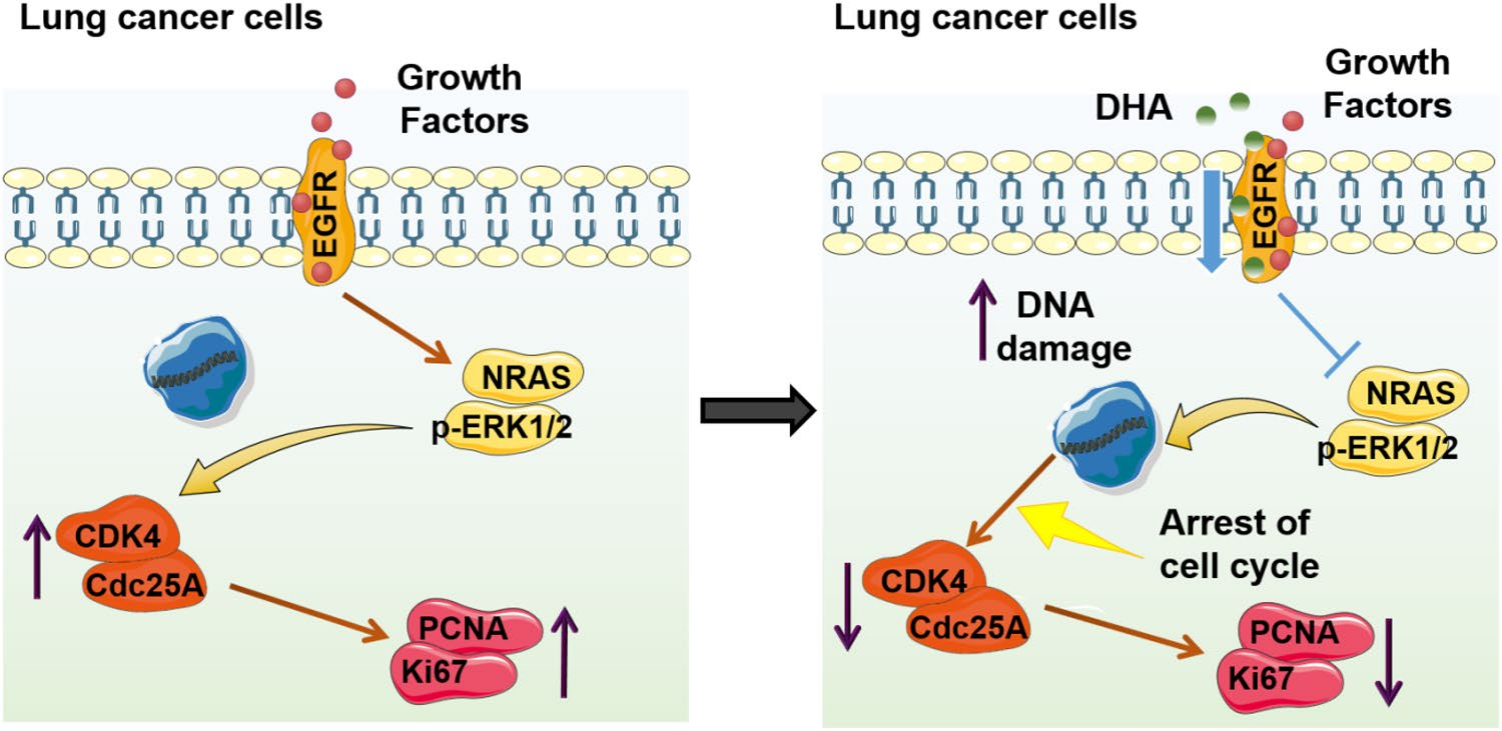
The diagram of the present work. 1. NRAS signaling pathway inhibited DNA damage and thereby facilitates the proliferation of lung cancer cells. 2. DHA could bind to EGFR molecules on the lung cancer cell membranes to drive the inhibitory effect. 3. DHA-driven blockage of the EGFR-NRAS signaling pathway leads to DNA damage, suppressing the proliferation of lung cancer cells thereupon.

The current work discovered that malignant lung cancer cells proliferated rapidly compared with normal lung cells due to the high expression of NRAS. DHA binds the EGFR and thereby dampens EGFR-NRAS signaling, leading to enhanced DNA damage and inhibiting cell cycle-mediated proliferation selectively in lung cancer cells (Fig. 9).

Molecular targeting therapy is a crucial strategy for precision tumor treatment that has emerged in recent years. It can achieve selective anti-tumor efficacy without affecting normal tissue cells by interfering with certain unique molecules, which appear in malignant cells but have no (or low) expression in normal cells. The Chinese medicine monomer DHA is low toxic, safe, and effective, and is an important scientific achievement achieved by the cross-fertilization of traditional Chinese medicine and modern medicine, which has chemotherapeutic effects on both plasmodium and malignant cells. In the present study, we further explored the novel mechanism of its effect on intracellular NRAS signaling pathways and DNA damage in lung cancer cells (Fig. 5), which provides both a basis for the translational application of DHA and a target intervention in anti-tumor molecular targeting therapy.

Interestingly, inhibition of the NRAS signaling pathway in lung cancer cells by DHA was accompanied by an intensification of DNA damage, which on the one hand led to apoptosis, as confirmed by several studies and our previous work^33,35–37^. However, on the other hand, DNA damage also resulted in cell cycle arrest, which can buy time for cell repair. Our results indicated that DHA treatment of lung cancer cells primarily arrested the cell cycle in the G1 phase, which was evidenced by a decrease in the expression of proteins and the kinase (CDK4 and CyclinD1) that regulate the progression of the G1 phase to the S phase. This is also an essential discovery of the present study. DHA-induced cell cycle arrest has resulted in a significant decrease in the rate of cell proliferation thereupon.

In the present research, to prove that the NRAS pathway was at the heart of the selective anti-proliferation effect of DHA on lung cancer cells, a reverse demonstration was carried out using the NRAS over-expression plasmid. Indeed, when NRAS was over-expressed, the DHA-induced DNA damage, cell cycle arrest, and cell proliferation inhibition effects were attenuated synchronously. Previous reports have shown that DHA was more effective in tumors with EGFR or NRAS mutations^38^. We considered that DHA may have an inhibitory effect on NRAS signaling thereupon. The targeting of DHA and EGFR is, of course, the primary source of the inhibition of NRAS signaling that drives DNA damage, which has never been reported before and is an important discovery (Fig. 6).

In a nutshell, the high expression of oncogene NRAS leads to the rapid proliferation of lung cancer cells. DHA can enhance DNA damage by uniquely banding to EGFR and thereby blocking the NRAS signaling pathway to achieve selective anti-lung cancer efficacy (Fig. 9). This work re-examined the new role and mechanism of DHA, a compound of Chinese phytomedicine, in the treatment of malignant tumors, especially NRAS mutated lung cancer, from the perspective of molecular targeting, proposing novel theoretical basis and feasible strategies for the application of Chinese medicine in molecular targeting therapy.

### Supplementary Information

Supplementary Information.

## Data Availability

The datasets used and/or analyzed during the current study are available from the corresponding author upon request.

## Abbreviations

BSA: Bovine serum albumin
CDK: Cyclin-dependent kinases
CETSA: Cellular thermal shift assay
DHA: Dihydroartemisinin
DDR: DNA damage response
EGFR: Epidermal growth factor receptor
IHC: Immunohistochemistry
LLC: Lewis lung cancer cells
LMPA: Low melting point agarose
MFI: Mean fluorescent intensity
WB: Western blotting
CO-IP: Co-immunoprecipitation
