# Supplementary material for: Dihydroartemisinin-driven selective anti-lung cancer proliferation by binding to EGFR and inhibition of NRAS signaling pathway-induced DNA damage
Source: Sci Rep. 2024 May 22;14:11704. doi: 10.1038/s41598-024-62126-8 (PMC11111767; doi:10.1038/s41598-024-62126-8)
Supplement: Supplementary file 1 — Supplementary Information. [file 41598_2024_62126_MOESM1_ESM.pdf]

# Supplementary Information

| Compounds and proteins | binding affinity | H-Bond sites |
|---|---|---|
| CID_3000518 (Dihydroartemisinin)<br>PDB code:3IKA (EGFR) | -7.3 kcal/mol | GLU-762, GLU-758, LYS-860 |
| CID_3000518 (Dihydroartemisinin)<br>PDB code:3IKA (EGFR) | -7.2 kcal/mol | TYR-869, ARG-836 |
| CID_3000518 (Dihydroartemisinin)<br>PDB code:3IKA (EGFR) | -7.1 kcal/mol | ARG-841 |
| CID_3000518 (Dihydroartemisinin)<br>PDB code:3IKA (EGFR) | -7.0 kcal/mol | Without H-bond sites |

**Table S1.** The detailed information of the molecular docking in **Fig. 6C-E**. The highest binding affinity of DHA and EGFR was -7.3 kcal/mol, wherein the possible sites of H-Bond were GLU-762, GLU-758, and LYS-860. Some other bindings sites of EGFR with the binding affinity were also listed in the table.

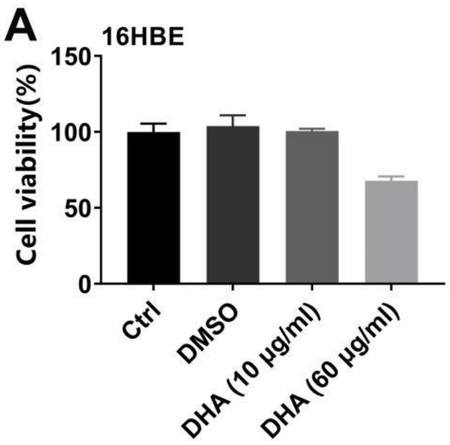


**Figure S1.** The cell viability of 16HBE treated by DHA was assayed by CCK-8 (n=3, * $p < 0.05$).

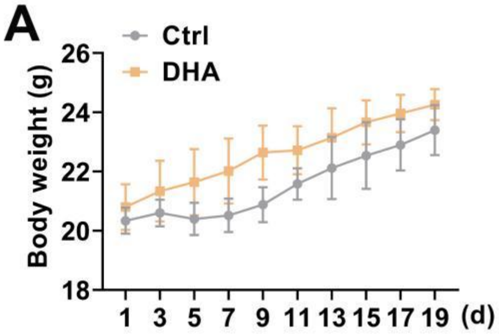


**Figure S2.** The body weight of tumor-bearing mice in the DHA treatment was monitored (n=5).

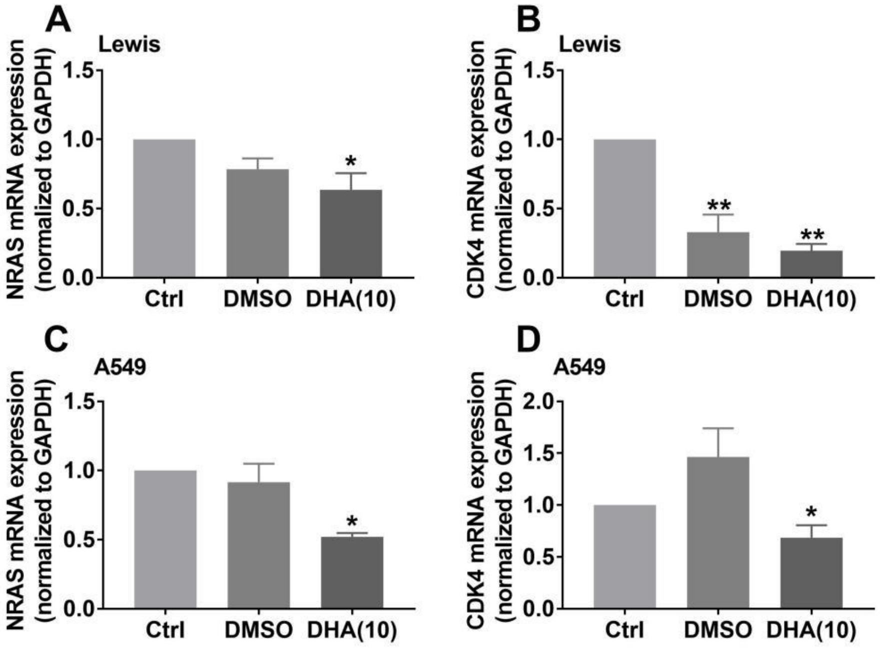


**Figure S3.** qRT-PCR assay of NRAS and CDK4 expression in DHA-treated Lewis and A549 cells (n=3, * $p < 0.05$).

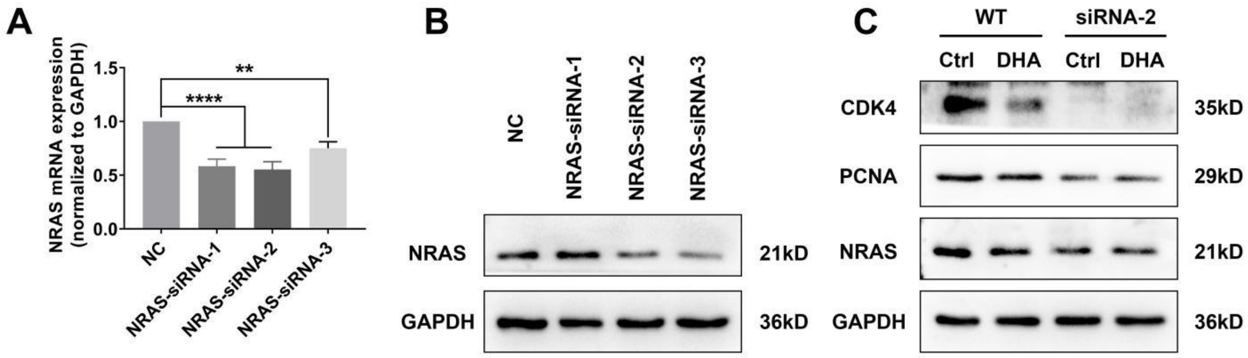


**Figure S4.** The NRAS in Lewis cells was knocked down with siRNA-NRAS. The knockdown efficiency of siRNA was validated by qRT-PCR and WB (A-B). The expression of NRAS, CDK4, PCNA was detected by WB in Lewis cells treated by siRNA2-NRAS and DHA (60 μg/ml) (C). The NRAS, PCNA, and CDK4 expression varied little in the NRAS knockdown groups.

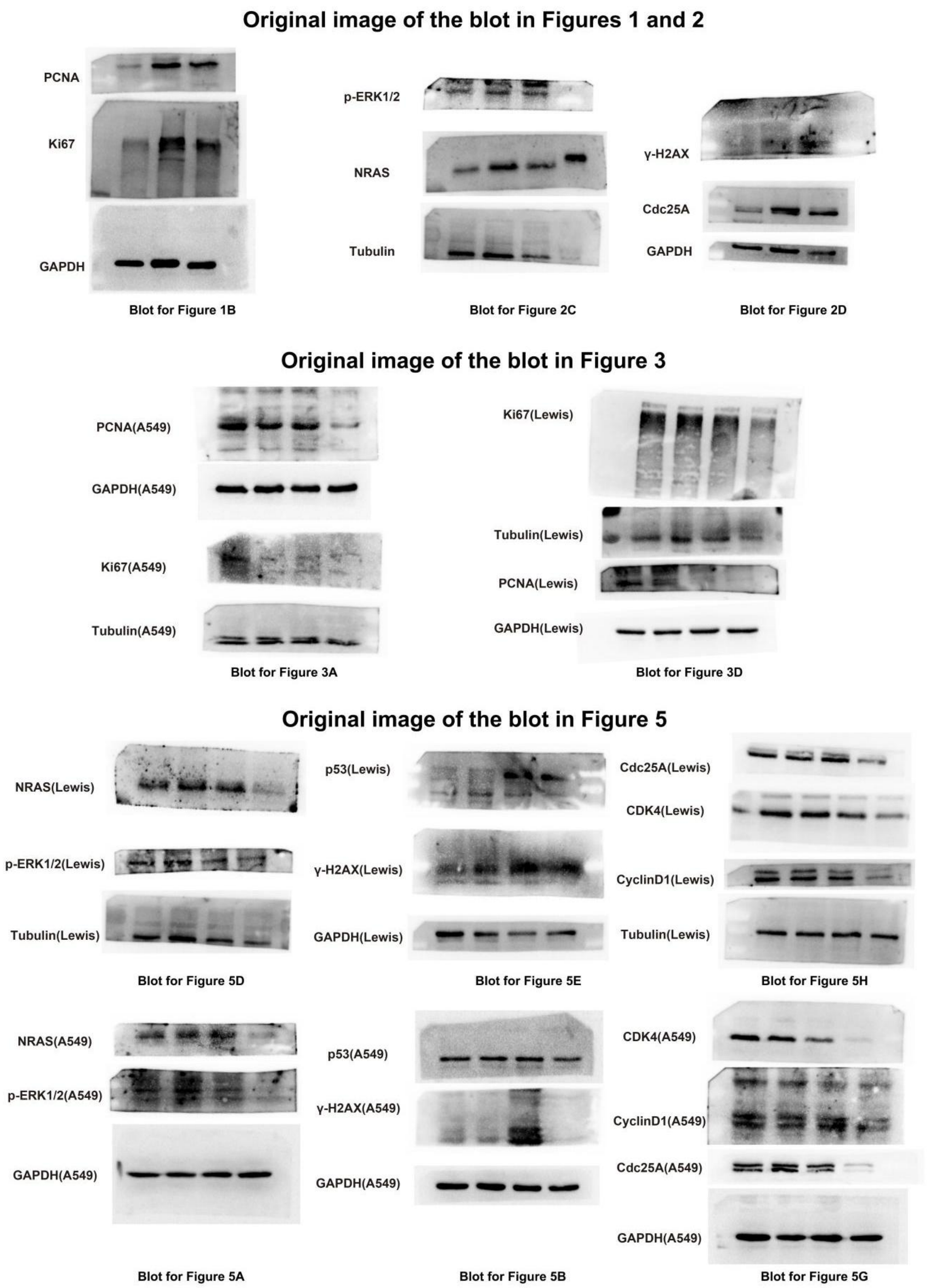


**Fig. S5.** Original bands of WB in the present work (Fig. 1-3 and Fig. 5).

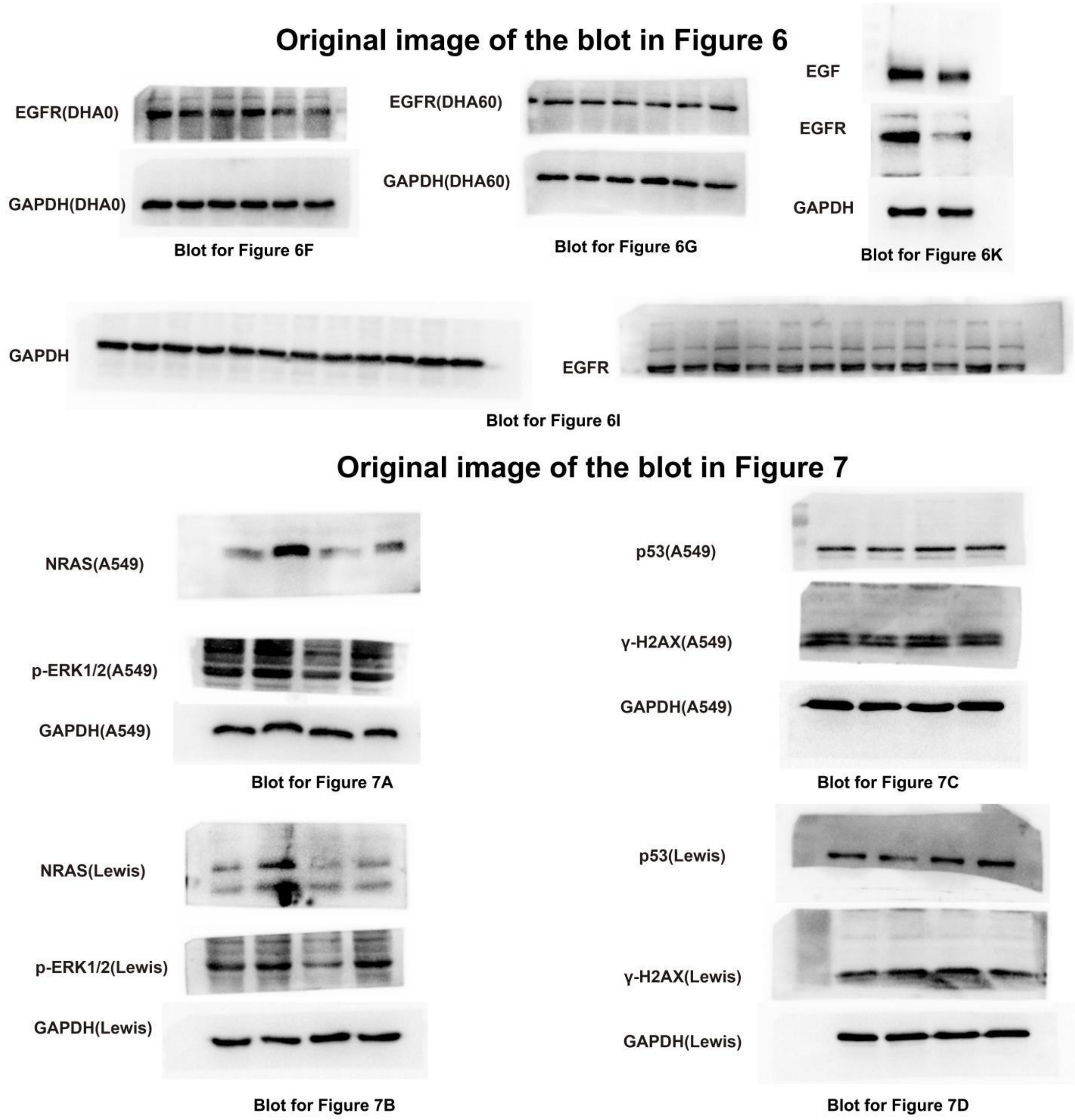


**Fig. S6.** Original bands of WB in the present work (Fig. 6-7).

## Original image of the blot in Figure 8

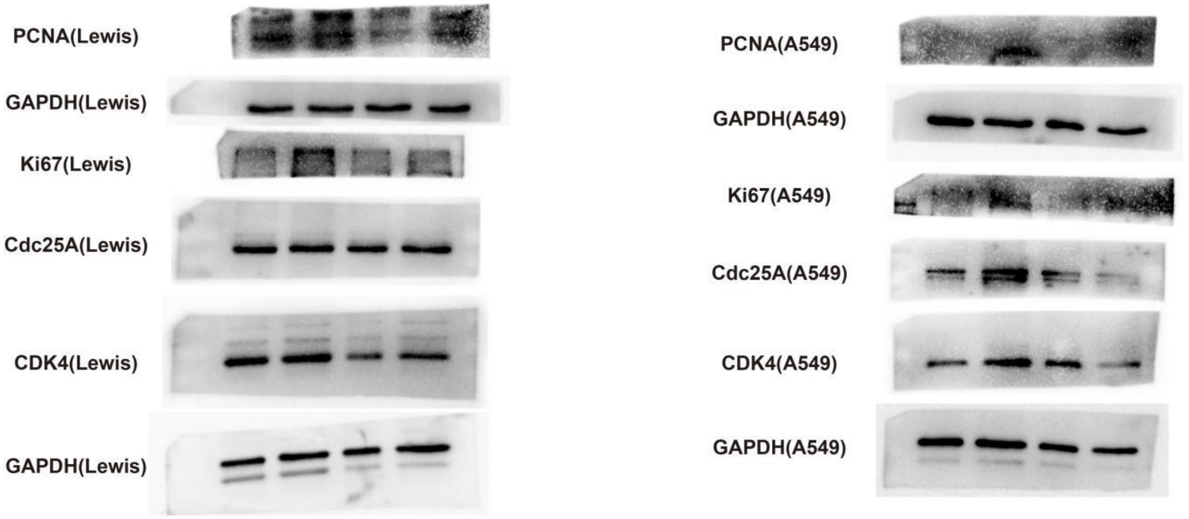


Blot for Figure 8B

Blot for Figure 8C

## Original image of the blot in Figure S4

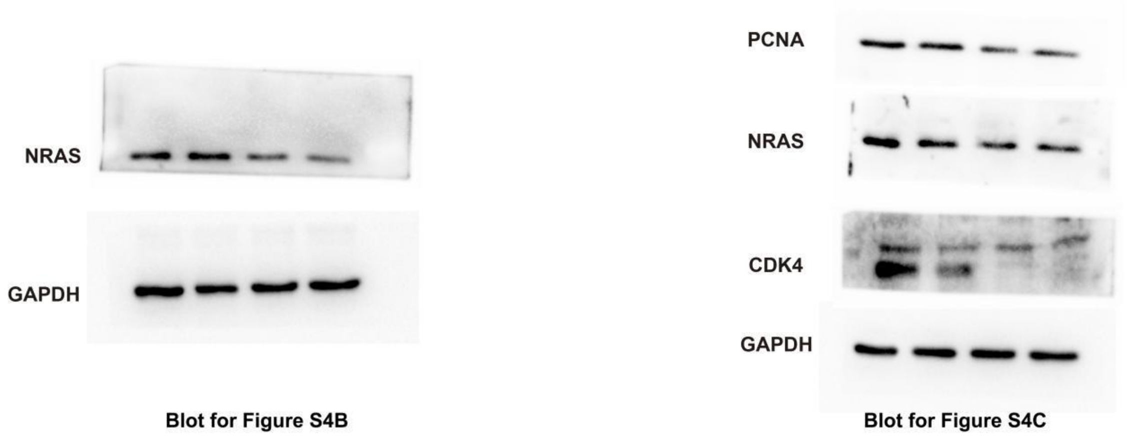


Blot for Figure S4B

Blot for Figure S4C

**Fig. S7.** Original bands of WB in the present work (Fig. 8 and S4).
